# Based on network pharmacology and molecular docking to explore the potential mechanism of shikonin in periodontitis

**DOI:** 10.1186/s12903-024-04618-7

**Published:** 2024-07-24

**Authors:** Qingliang Zhao, Kun Wang, Lin Hou, Lin Guo, Xiangyan Liu

**Affiliations:** 1 Department of Stomatology, Harbin the First Hospital, Harbin, 150010 China; 2 Department of Central Sterile Supply, the First Affiliated Hospital, Harbin Medical University, Harbin, 150001 China; 3 School of Integrative Medicine, Tianjin University of Traditional Chinese Medicine, 10 Poyanghu Road, West Area, Tuanbo New Town,Jinghai District, Tianjin, 301617 China

**Keywords:** Shikonin, Periodontitis, Network pharmacology, Molecular docking, Molecular mechanism

## Abstract

**Objectives:**

To investigate the potential mechanisms of shikonin in preventing and treating periodontitis using network pharmacology and molecular docking methods.

**Materials and methods:**

The targets of shikonin were obtained in TCMSP and SEA databases, and targets of periodontitis were gathered from the OMIM, GeneCards and Drugbank Databases. The intersecting targets were entered into the DAVID database to obtain the relevant biological functions and pathways by GO and KEGG enrichment analysis. The obtained targets were analysed the protein–protein interaction (PPI) in STRING platform. In Cytoscape 3.8.0, the network analysis function with the MCODE plug-in were used to obtain the key targets, of shikonin and periodontitis. Molecular docking and molecular dynamics simulation (MD) were used to assess the affinity between the shikonin and the key targets.

**Results:**

Shikonin was screened for 22 targets and periodontitis was screened for 944 targets, the intersecting targets were considered as potential therapeutic targets. The targets played important roles in cellular response to hypoxia, response to xenobiotic stimulus and positive regulates of apoptotic process by GO enrichment analysis. 10 significant pathways were analyzed by KEGG, such as human cytomegalovirus infection and PI3K-Akt signaling pathway, etc. Cytoscape software screened the key genes including AKT1, CCL5, CXCR4, PPARG, PTEN, PTGS2 and TP53. Molecular docking and MD results showed that shikonin could bind stably to the targets.

**Conclusions:**

The present study enriched the molecular mechanisms in periodontitis with shikonin, providing potential therapeutic targets for periodontitis.

**Supplementary Information:**

The online version contains supplementary material available at 10.1186/s12903-024-04618-7.

## Introduction

Periodontitis, an inflammatory disease in periodontal tissues, is a chronic infectious disease which influenced by multiple factors. In the 2019 Global Burden of Disease (GBD) Study, the global prevalence, age-standardised rate (ASR) and estimated annual percentage changes (EAPCs) of periodontitis showed an increasing trend [1, 2]. There were 1,087,367,744.0 cases (including 91,518,820.6 new cases) of periodontitis globally in 2019 [2]. China's fourth national oral health survey showed that the prevalence of periodontal disease among people over 35 years was 82.6%—88.4% [3]. Therefore, periodontitis is a globally important public health problem, and its prevention and treatment are urgent. Periodontitis is the invasion of gingival tissues with bacteria and periodontal pathogens [4]. Periodontitis is referred to as a ‘mixed bacterial infection’, and it often leads to gum tissue damage and tooth loss [5, 6]. The common causative organisms including porphyromonas gingivalis [7], tannerella forsythia [8], treponema denticola [9], fusobacterium nucleatum [10] and aggregatibacter actinomycetemcomitans [11], which produce vasoactive amines [12], tumor necrosis factor-alpha (TNF-α), Interleukin-1β (IL-1β), prostaglandin E2 and matrix metalloproteinases (MMPs). Then the factors lead to irreversible destruction of the alveolar bone and periodontal ligaments [13]. In addition, periodontitis is an important risk factor for other systemic diseases [14], such as diabetes [15], cardiovascular disease [16], non-alcoholic fatty liver disease [17], Alzheimer's disease [18], cancer [19]. During periodontal infection, pathogenic microorganisms can easily spread into the circulation through the ulcerated epithelium of the deep periodontal pockets, affecting other tissues and organs [20, 21]. People with periodontitis are more likely to suffer from heart disease, hypertension, atherosclerosis and immune diseases (arthritis) [20]. Periodontitis also increases mortality in chronic kidney disease. Numerous studies have found that periodontitis increases the incidence of cancer. For example, periodontitis reduce alveolar bone 1 mm will lead to a fivefold increase in the incidence rate of tongue cancer [21]. Common clinical symptoms of periodontitis including swollen and bleeding gums, toothache, loose and shifting tooth, and even tooth loss [13]. The treatments for periodontitis including basic, surgical and pharmacological treatments [12, 22]. Basic treatment consists of oral hygiene instructions [23], supragingival scaling [24], subgingival scaling [25] and root planing [26]. Surgical treatments include flap procedures [27, 28], gingivoplasty [28, 29], gingivectomy [28], and guided tissue regeneration [30]. Since surgical treatment requires to considerate the tolerance of patients. Drugs are uesd to reduce and eliminate the infection in periodontitis [31], with antibiotics [32] and non-steroidal anti-inflammatory drugs [33]. However, antibiotics and other drugs are often associated with resistance and adverse effects, such as gastrointestinal tract dysfunction and the damage in liver and kidney [34, 35]. The use of natural compounds (natural medicines) is a viable treatment option for periodontitis as they have lower adverse effects. In addition, the natural compounds have shown great therapeutic effects in multiple diseases [36–38].

Shikonin (SHI) is an active ingredient from a traditional Chinese medicinal herb: *Lithospermum erythrorhizon. *Shikonin has be well-defined anti-microbial [39, 40], anti-inflammatory [41] and anti-tumour effects [42–44]. It was found that shikonin reduced the expression of IL-1β, IL-6 and TNF-α in the human periodontal ligament cells (hPDLCs) inflammation model [45]. Furthermore, shikonin can inhibit osteoclast differentiation, enhance bone mesenchymal stem cells, achieve bone regeneration and repair bone defects in periodontitis [46]. Numerous studies found that shikonin had beneficial effects in periodontitis, but its specific mechanism need to be further explored.

Network pharmacology integrates systems biology, pharmacology, computer-based virtual laboratory simulations and network topology analysis to assess the pharmacological effects at multiple levels and directions [47, 48]. In order to obtain relevant targets and potential mechanisms of diseases and drugs, network pharmacology is widely used to assist the research in various diseases [49, 50]. Molecular docking is a computer simulation technique which can explain the interactions between ligands and proteins [51–53]. In this study, the potential mechanisms and targets of shikonin in periodontitis were analysed by network pharmacology, molecular docking and MD.

## Materials and methods

### Gene dataset acquisition of shikonin and periodontitis

Target genes of shikonin were gathered from two databases: Traditional Chinese Medicine Systems Pharmacology Database and Analysis Platform Database (https://tcmsp-e.com/) and Similarity Ensemble Approach (SEA) Database (https://sea.bkslab.org/). Target genes of periodontitis were gathered from three databases, including the Online Mendelian Inheritance in Man Database (http://www.omim.org/), GeneCards Database(https://www.genecards.org/) and Drugbank Database (https://go.drugbank.com) with “Periodontitis” as the keyword.

### GO analysis and KEGG pathway enrichment analysis

DAVID Bioinformatics Database (https://david.ncifcrf.gov) have a variety of functions, such as gene annotation, online analysis, functional enrichment, interactive analysis, etc. It also contains the Kyoto Encyclopedia of Genes and Genomes (KEGG) database and GO database. The analysis of KEGG can provide a access for biological functions and relevant candidate targets. GO enrichment can analysis the biological process (BP), molecular function (MF) and cellular component (CC). The targets of shikonin and periodontitis were imported into the DAVID database, and "Homo sapiens" was used as the selected condition to obtain the biological functions and important targets.

### Construction of protein–protein interaction network

The STRING database (https://string-db.org/) bases on their network topological features to provide a intuitive access for the dynamic network of proteins. shikonin and periodontitis targets were imported into an online Veen diagram platform to obtain the overlapping genes. The obtained target genes were imported into the STRING platform to analyse the protein–protein interaction (PPI) with the "Homo sapiens" and confidence level of 0.9 as the screening condition.

### Network construction

Cytoscape software performs visual analyses to obtain inner associations between genes based on network topology features [54]. Genes obtained by Cytoscape software may be pivotal genes for shikonin to treat periodontitis.

### Molecular docking

Molecular docking can predict the binding mechanism and activity between molecules and relevant targets [55]. The molecular structure of shikonin was obtained from the PubChem database (https://pubchem.ncbi.nlm.nih.gov/). Protein kinase B (AKT1, PDB ID: 6HHH), C–C chemokine ligand 5 (CCL5, PDB ID: 6AEZ), C-X-C chemokine receptor type 4 (CXCR4, PDB ID: 3ODU), Peroxisome proliferator-activated receptor gamma (PPARG, PDB ID: 6FZG), Phosphatase and tensin homolog (PTEN, PDB ID: 1D5R), Prostaglandin-endoperoxide synthase 2 (PTGS2, PDB ID: 5F19) and Cellular tumor antigen p53 (TP53, PDB ID: 7LIN) in PDB format were obtained from the Protein Data Bank (https://www.rcsb.org/). BIOVIA Discovery Studio Visualizer 2021 was used to screening active ingredients and preparing proteins [56]. After removing the crystalline water molecules and replenishing incomplete amino acid residues, using LibDockScore to assess the affinity between the shikonin and the targets. In addition, Pymol software [57], a sub-software of python software, was used to process and analyse the molecular docking results [55, 58].

### Molecular dynamic simulation

Molecular dynamics simulation (MD) was perfored by GROMACS 2023 software package [59]. The protein and ligand used the CHARMM 36 and GAFF2 force field parameter. Using periodic boundary conditions, the protein–ligand complexes were placed in a cubic box with a boundary of 12 Å, and water molecules were filled into the box using the TIP3P water model. Using the steepest descent method, the system was be optimized at 50000 steps. The system was gradually heated in NVT ensemble, then subjected to NPT. Van-der-Waals and Coulomb interactions were calculated(1.0 nm). 100 ns molecular dynamics simulations were performed with a time step of 2 fs (constant temperature:300 K and constant pressure:1 bar) [60].

## Results

### Gene targets of shikonin

Twenty-two targets were obtained from the TCMSP database, and the SEA database retrieved 10 target genes. A total of 22 target genes was identified (Supplementary Table 1: The 22 target genes of shikonin).

### Gene targets of periodontitis

Target genes of periodontitis were gathered from three databases, 11 in OMIM, 935 in GeneCards Database and 14 in Drugbank Database. A total of 944 gene targets were identified in periodontitis (Supplementary Table 2: The 944 genes were associated with periodontitis).

### GO and KEGG enrichment analysis

**Fig. 1:**
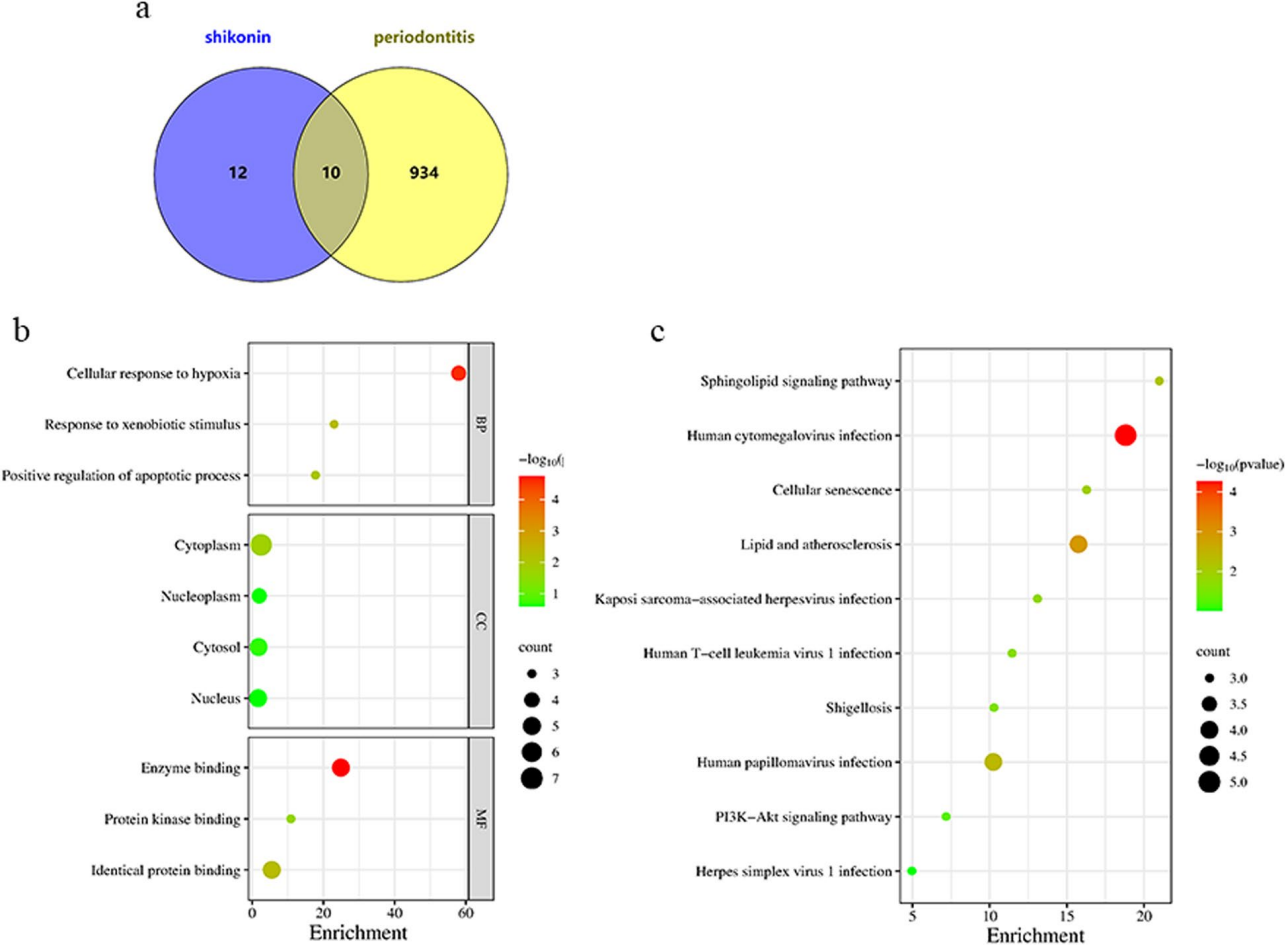
GO and KEGG enrichment analysis of shikonin and periodontitis. **a** overlapping genes of shikonin and periodontitis. **b** GO enrichment analysis of the overlapping genes in shikonin and periodontitis. **c** KEGG pathway analysis of the overlapping genes in shikonin and periodontitis

The common target genes of shikonin and periodontitis were imported into the DAVID database for GO and KEGG analysis (Fig. 1a). The respective top enriched terms of BP, CC, and MF were presented in Fig. 1b. The results of GO enrichment analysis indicated that the targets were involved in many important biological functions. In the biological process category, targets played important roles in cellular response to hypoxia, response to xenobiotic stimulus and positive regulates of apoptotic process. In the cellular component category, including enzyme binding, identical protein binding and protein kinase binding. We performed KEGG pathway analysis to retrieve 10 significant pathways (*P* < 0.05), such as human cytomegalovirus infection, lipid and atherosclerosis, human papillomavirus infection and PI3K-Akt signaling pathway, etc. (Fig. 1c). Based on the above analysis, we knowed that the treatment of shikonin in periodontitis may be associated with inhibition of inflammation.

### Construction of PPI network and cytoscape network

**Fig. 2:**
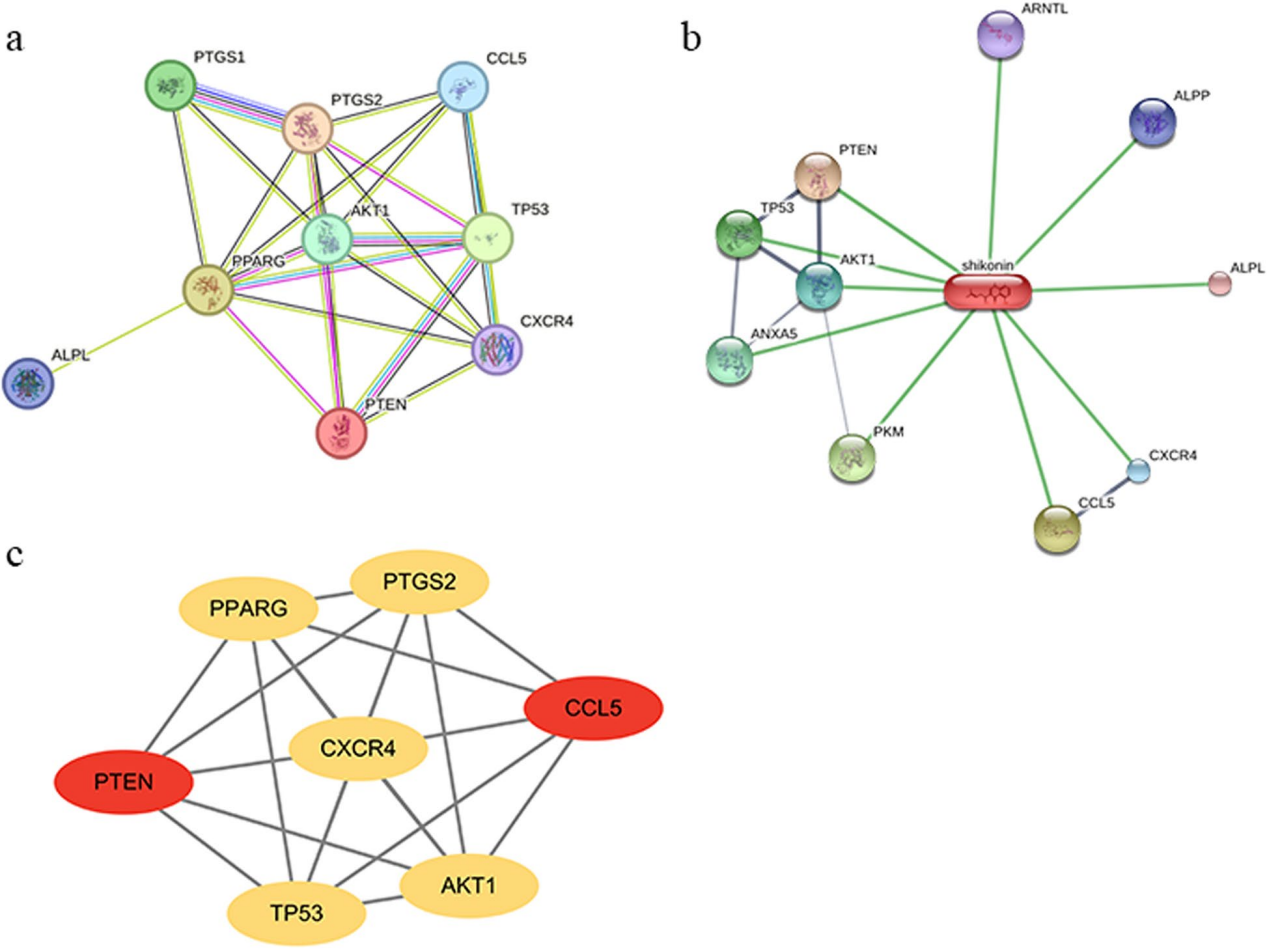
PPI network and Cytoscape network of shikonin and periodontitis. **a** the PPI network of shikonin and periodontitis genes. **b** Visual correlation diagram of shikonin with important proteins in PPI network. **c** Cytoscape analysis of key target genes with PPI network information

The overlapping genes of shikonin and periodontitis were imported into the STRING database to produce PPI network and obtain protein information (Fig. 2a and b, Table 1). Based on the network topological features, the graph information in PPI network was imported into Cytoscape software to screen the key nodes, then revealing the key target genes including AKT1, CCL5, CXCR4, PPARG, PTEN, PTGS2 and TP53 (Fig. 2c).

**Table 1:** Characteristics of the genes in PPI network

Gene	Name	Degree
AKT1	Protein kinase B	7
ALPL	Tissue-nonspecific alkaline phosphatase	1
ALPP	Placental alkaline phosphatase	0
CCL5	C–C chemokine ligand 5	5
CXCR4	C-X-C chemokine receptor type 4	6
PPARG	Peroxisome proliferator-activated receptor gamma	8
PTEN	Phosphatase and tensin homolog	5
PTGS1	Prostaglandin-endoperoxide synthase 1	3
PTGS2	Prostaglandin-endoperoxide synthase 2	7
TP53	The tumor suppressor p53	6

### Molecular docking

**Fig. 3:**
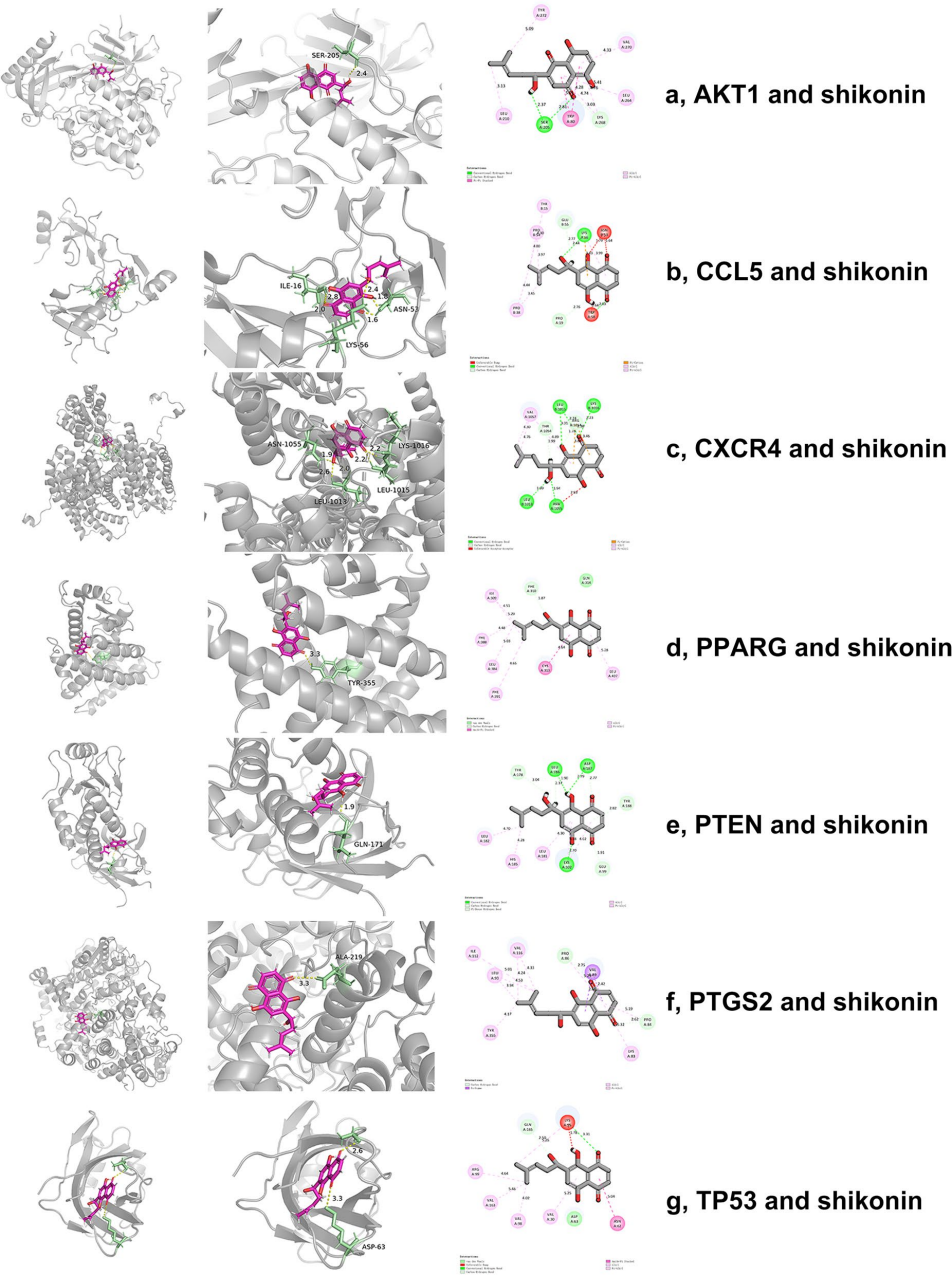
Molecular docking diagrams of related targets in shikonin and periodontitis. **a** Molecular docking of AKT1 and shikonin. **b** Molecular docking of CCL5 and shikonin. **c** Molecular docking of CXCR4 and shikonin. **d** Molecular docking of PPARG and shikonin. **e **Molecular docking of PTEN and shikonin. **f **Molecular docking of PTGS2 and shikonin. **g **Molecular docking of TP53 and shikonin

To validate the findings of Network Pharmacology, molecular docking was used to assess the binding relationship between shikonin and the targets. The 7 targets (AKT1, CCL5, CXCR4, PPARG, PTEN, PTGS2, TP53) obtained from the PPI network and Cytoscape, and the docking results were shown in Table 2 and Fig. 3. Shikonin bound to AKT1 (6HHH) by 1 hydrogen bonds between it and SER-205. Shikonin bound to CCL5 (6AEZ) by 4 hydrogen bonds between it and ILE-16, LYS-56 and ASN-53. Shikonin bound to CXCR4 (3ODU) by 5 hydrogen bonds with ASN-1055, LEU1013, LEU1015 and LYS-1016. Shikonin bound to PPARG (6FZG) by 1 hydrogen bonds between it and TYR-355. Shikonin bound to PTEN (1D5R) by 1 hydrogen bonds between it and GLN-171. Shikonin bound to PTGS2 (5F19) by 1 hydrogen bonds between it and ALA-219. Shikonin bound to TP53 (7LIN) by 2 hydrogen bonds between it and ASP-63. The binding energies of ligand and receptors were -6.37 kcal/mol (AKT1), -6.12 kcal/mol (CCL5), -3.89 kcal/mol (CXCR4), -4.23 kcal/mol (PPARG), -4.25 kcal/mol (PTEN), -4.55 kcal/mol (PTGS2), -4.13 kcal/mol (TP53). It is generally accepted that binding energies less than -1.19 kcal/mol indicate that docking between ligand and receptor is feasible [61]. Binding energies less than -5 kcal/mol indicate good binding activity between ligand and receptor. Based on the docking results, we concluded that shikonin binds well to AKT1 and CCL5. The docking results were imported into PyMOL for preprocessing, as shown in Fig. 3.

**Table 2:** Docking simulation for active molecular and targets of shikonin in periodontitis

Molecular name	Targets	PDB ID	Residue involved in H bonding	H-bond length (Å)	Affinity (kcal/mol)
shikonin	AKT1	6HHH	SER-205	2.4	-6.37
shikonin	CCL5	6AEZ	ILE-16, LYS-56, ASN-53	2.8, 2.4, 1.8	-6.12
shikonin	CXCR4	3ODU	ASN-1055, LEU1013, LEU1015, LYS-1016	1.9, 2.6, 2.2, 2.2	-3.89
shikonin	PPARG	6FZG	TYR-355	3.3	-4.23
shikonin	PTEN	1D5R	GLN-171	1.9	-4.25
shikonin	PTGS2	5F19	ALA-219	3.3	-4.55
shikonin	TP53	7LIN	ASP-63	3.3	-4.13

### molecular dynamic simulation

**Fig. 4:**
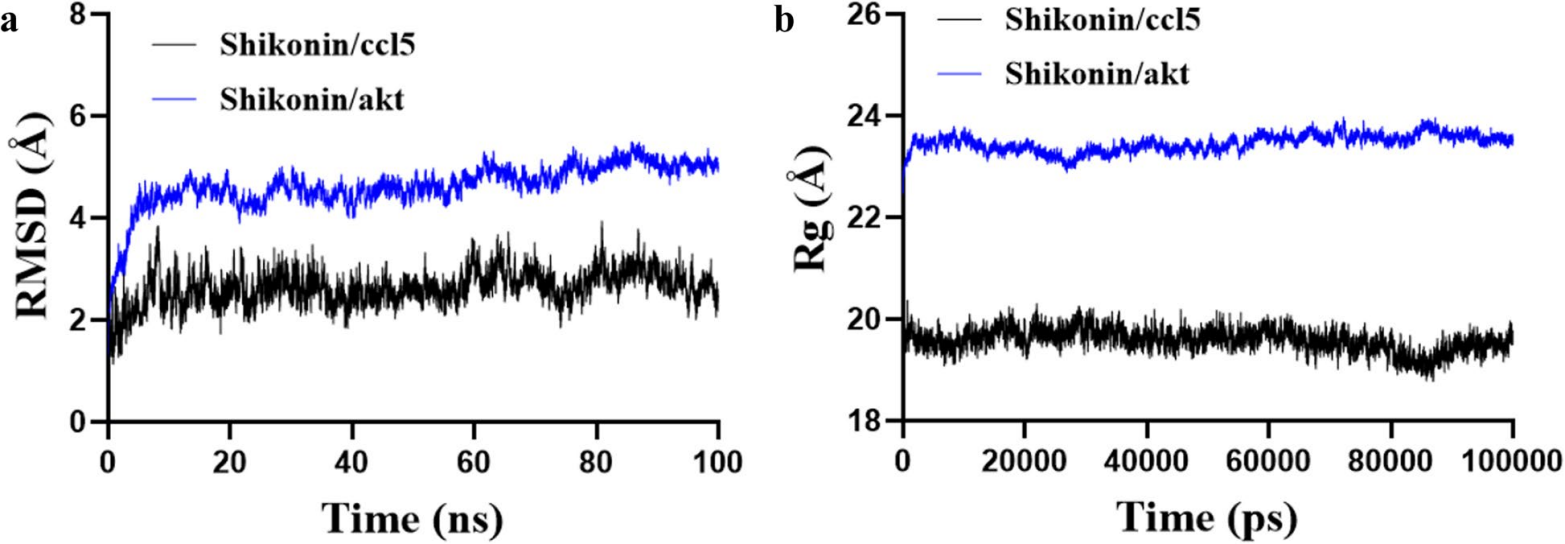
Molecular dynamics simulation (MD) of related targets in shikonin and periodontitis. **a **Time dependence of root mean square deviation (RMSD) of the complex shikonin/ccl5 and shikonin/akt. **b **Radius of gyration (Rg) of the complex shikonin/ccl5 and shikonin/akt

Based on the docking results, we concluded that shikonin has great binding ability with AKT1 and CCL5. We verified the binding ability between comedones and key target proteins by molecular dynamics simulation (MD). The equilibration of the simulation system was assessed using RMSD. The complex shikonin/CCL5 and shikonin/akt reached stability after about 10 ns. RMSD results also showed that shikonin/CCL5 has relatively high stability compared to shikonin/akt (Fig. 4a). In addition, the Radius of Gyration (Rg) can be used to describe the overall structural changes and the compactness of the protein structure. The shikonin/CCL5 complex had lower Rg than shikonin/akt (Fig. 4b). The results indicated that the Rg of shikonin/CCL5 is more likely to be stabilised at lower values and is more structurally stable. Therefore, the compound may directly bind to the CCL5 protein to exert its pharmacological effects.

## Discussion and future prospects

The Federation Dentaire Internationale (FDI) reported that periodontitis is a common chronic infectious disease with a global prevalence of 50 percent [62]. It is always known that severe periodontitis can lead to impaired chewing dysfunction, tooth loss and facial collapse [13, 63, 64]. In modern medicine, in order to manage and cure periodontitis, dentists should start from reducing the risk factors, suppressing the etiology and improving the treatment plan of periodontitis. In 2020, the European Federation of Periodobology (EFP) published clinical guidelines for the treatment of periodontitis including Stage I, Stage II. and Stage III. Mild to moderate cases of periodontitis (Stage I, II.) should be treated as early as possible, such as home care, scaling, root planing and antibiotic therapy [26, 65]. Severe cases of periodontitis, teeth with periodontal pocket depth (PD) ≥ 6 mm after Stage I and Stage II treatment, require surgical treatment and adjunctive systemic antibiotics [66–68].

Clear diagnosis, reduction of risk factors, improvement of home care education, and effective treatments are the keys of clinical prevention and treatment in periodontitis. The non-surgical treatment methods for periodontitis include home care education, tooth cleaning, root planing, and medication, which can improve the symptoms of periodontitis [69]. But there are some shortcomings in scaling, polishing, root planing, and drugs [70]. Frequent scaling and polishing can increase the sensitivity of teeth. Due to the effects of pacemakers, blood disorders and oral anticoagulants, scaling and root planing may be contraindicated for some people. In addition, there is an increased risk of infectious diseases if the healthcare environment is unsafe. Numerous studies have reported that drug therapy is also one of the main non-surgical treatments, mainly using antibiotics such as nitroimidazole, tetracycline, macrolide, and nonsteroidal anti-inflammatory drugs, which have significant therapeutic effects [69, 71, 72]. However, long-term drug treatment can also lead to drug resistance, dysbiosis, bone loss and other adverse reactions [70]. Chlorhexidine is a common clinical mouthwash that can reduce plaque, but it has some side effects [34]. For example, allergic reactions, tooth staining and harmful effects on host cells [34]. Surgical treatment can cause tooth loss and reduce tooth survival [73]. Removal of periodontal surgery may cause dentin hypersensitivity, food impaction, and aesthetic problems [74]. In addition, surgical treatment will also increase the incidence rate of oral cancer [21].

Pathogens infecting periodontal tissue or the oral cavity can stimulate T-cell activation, produce inflammatory factors, decrease leukocyte activity and increase the inflammatory response [75]. Therefore, eliminating or controlling the inflammation of periodontal tissues to promote cell homing and tissue repair is one of the main treatments for periodontitis [76]. Traditional Chinese medicine also has good effects in treating oral diseases such as periodontitis. In a clinical randomized controlled trial with oral mucosal inflammation, it was found that the traditional Chinese medicine group can significantly improve oral mucosal dryness, alleviate pain and inflammation [77]. Specific herbal mouthwashes also have an inhibitory effect on plaque and inflammation in periodontitis [78]. Numbers of natural compounds have been shown as low side effects and adverse effects [79–81]. A study reported that Trans-cinnamic aldehyde inhibited the inflammatory factors and reduced bone loss in periodontitis [82]. β-carotene can inhibit the progression of periodontitis by inhibiting nuclear factor kappa-B (NF-κB) activity and reducing the inflammatory cytokines [83]. Notopterol reduces the release of inflammatory mediators and reactive oxygen species in periodontitis by regulating NF-κB /Akt signaling [84, 85]. Resveratrol, psoralen and angelicin can reduce inflammation in periodontitis [86]. In addition, the combination of natural compounds and existing treatment methods can achieve better results in treating periodontitis, such as increasing anti-inflammatory and antibacterial effects, shortening treatment courses, and reducing recurrence rates. The combination of anthocyanins and Secnidazole can reduce the pathogenic bacteria in periodontitis [84]. The combination of curcumin and chlorhexidine can also alleviate symptoms in patients with periodontitis [87].

Shikonin is an active compound, and its anti-inflammatory effects have been demonstrated in the treatment of various diseases [88, 89]. The anti-inflammatory effects of shikonin in periodontitis have also received a lot of attention. In 2016, Satoru Shindo et al. [39] demonstrated that shikonin had a therapeutic effect on periodontitis for the first time. In vitro*,* a periodontitis model was constructed by stimulating HPDLC with IL-1β and TNF-α, and shikonin significantly reduced the expression of IL-6, IL-18, and cysteine-cysteine motif chemokine ligand 20 (CCL20) [39]. A study also found that the high levels of inflammatory cytokines, matrix metalloproteinase-2 (MMP-2), MMP-9, and cyclooxygenase-2 (COX-2) produced by LPS stimulation in hPDLC were also reversed by shikonin [45]. In addition, shikonin can promote the proliferation and migration of human gingival fibroblasts (hGF), stimulate the expression of osteopontin (OPN) and osteogenic differentiation to repair bone and periodontal tissues [90]. A study also reported that Shikonin can act as a specific inhibitor of PKM2, inhibiting osteoclastogenesis and alleviating bone loss in periodontitis [91].

The efficacy of shikonin in periodontitis has been well reported, however, the specific mechanisms are not clear. In this study, network pharmacology was used to explore the relationship between shikonin and periodontitis. The KEGG enrichment analysis was used to enrich for targets of shikonin and periodontitis, and obtained that the signalling pathways which related with inflammatory and infectious diseases. The PPI network was analysed by Cytoscape and obtained key targets, including AKT1, CCL5, CXCR4, PPARG, PTEN, PTGS2 and TP53. The relationships between shikonin and key targets were verified by molecular docking. Shikonin can treat rheumatoid arthritis [92], kidney injury [93], cancer [94],and myocardial ischaemia–reperfusion injury [95] by inhibiting the PI3K-Akt signalling pathway. It was found that shikonin can induce apoptosis in Burkitt's lymphoma by inhibiting the phosphatidylinositide 3-kinase (PI3K)/AKT/mammalian target of rapamycin (mTOR) pathway [96]. Shikonin also protected the dental pulp by stimulating differentiation of dental pulp stem cells (DPSCs) via the AKT-mTOR signalling pathway [97]. Shikonin inhibited the expression of CCL5 [98] and CXCR4 [99] and suppressed the proliferation of neutrophils in chronic granulocytic leukaemia. Shikonin attenuated oxidative stress, apoptosis,and neuroinflammation in Parkinson's disease [100] and renal impairment disorders [93] by the Akt/Extracellular regulated protein kinases (ERK)/c-Jun N-terminal kinase (JNK)/NF-κB pathway. A Study reported that shikonin may act as an antagonist of PPARγ to inhibit lipogenesis [101], which involved in the treatment of type 2 diabetes [102], fatty liver disease [103] and obesity. LPS induced the upregulated expression of PTEN and p-AKT, but shikonin reversed the phenomenon [104, 105]. Furthermore, It had been demonstrated that the key targets (AKT1 [106], CCL5 [107], CXCR4 [108], PPARG [109], PTEN [106], PTGS2 [110, 111] and TP53 [112, 113]) had a close association with periodontitis.

As is well known, traditional Chinese medicine works on diseases through multiple targets and pathways. We topologically analyzed on the protein–protein interaction network using Cytoscape to construct a network (active ingredients-common targets) [114]. Combining literature and KEGG Map information, CCL5 and AKT are correlated with inflammatory responses in the toll-like receptor signaling pathway (map04620). The virus binds to CCL5 or CXCR4 receptors, activating downstream AKT pathways, leading to inflammation or apoptosis reactions (map05170). It was found that cordycepin attenuated inflammation and apoptosis by inhibiting the Akt/NF-κB signaling pathway mediated by CCL5 [115]. There is also a positive correlation between CCL5 and Akt in microglia and macrophages [116]. CXCR4 has been found to be an important inflammatory regulator in periodontitis, inhibiting inflammation and reducing pain by regulating the PI3K/Akt [117] and Akt/NF-κB [118] pathway. Bavachinin and cardamonin are natural components of traditional Chinese medicine. Bavachinin can alleviate inflammation, apoptosis, and oxidative stress in periodontitis by regulating PPARG/PI3K/AKT/ PTEN signaling [119, 120]. Cardamonin also has an anti-inflammatory effects on HPDLC,which was mediated by reducing the expression of CCL5 and PTGS2 [121]. In addition, it has been found that the correlation of Akt with PTGS2 in animal and cell studies of periodontitis [122, 123]. It can be seen that the targets analyzed by Cytoscape also have significant correlations with each other. Therefore, we speculate that shikonin can improve the periodontitis by targeting multiple targets.

Based on the docking results, we concluded that shikonin has great binding ability with AKT1 and CCL5. Based on the MD results, the complex shikonin/CCL5 and shikonin/akt reached stability after about 10 ns. RMSD results also showed that shikonin/CCL5 has relatively high stability compared to shikonin/akt. The shikonin/CCL5 complex had lower Rg than shikonin/akt. Therefore, we hypothesised that CCL5 and AKT are the targets of shikonin in periodontitis.

Although shikonin has not yet been applied in the clinical treatment of periodontitis, it has been widely used in other diseases. Clinical studies have found that shikonin can improve the symptoms of mild to moderate diaper dermatitis in children [124]. Shikonin can reduce the expression of serum inflammatory markers TNF-α in psoriasis patients and improve the skin barrier function [125, 126]. Shikonin also has therapeutic effects on wound healing and inflammatory reactions after anal fistula surgery [127]. Numerous clinical studies have confirmed that shikonin is safe and beneficial for clinical treatment. Therefore, there is also potential for the use of shikonin in periodontitis.

Periodontitis is an important oral disease for human health. Existing treatments have significant efficacy, but some side effects (such as drug resistance, imbalance of intestinal flora and adverse reactions) are accompanied. In recent years, Chinese herbs (natural compound components) have attracted attention for good therapeutic effects in periodontitis through multiple targets and pathways [81]. In clinical, animal and cellular studies, it has been found that shikonin has good anti-inflammatory effects in various diseases. In particular, shikonin also can promote hGF proliferation and migration, osteogenic differentiation. Therefore, the definite mechanism for shikonin needs to be explored in periodontitis. The results of this study enrich the pathways of shikonin in periodontitis, and identify potential targets (AKT1, CCL5, CXCR4, PPARG, PTEN, PTGS2 and TP53) for its anti-inflammatory effects. Performing molecular docking and molecular dynamic simulation to verify the results. A study have shown that when drugs (natural compounds) are incorporated into nanoparticle carriers can increase their antimicrobial activity in periodontitis [86]. Therefore, we speculate that natural compounds (shikonin) combine nanotechnology can bring good news for patients with periodontitis in the future. The present study enriches the connotation and the potential mechanism of shikonin in periodontitis. We will conduct experimental verification for our results in the future. Meanwhile, the results (targets) obtained in this study may be an important pivot in the prevention and treatment of periodontitis, in order to provide a rich foundation for future clinical treatment of periodontitis.

## Conclusion

In this study, we investigated the mechanisms of shikonin in periodontitis and obtained the potential targets, including AKT1, CCL5, CXCR4, PPARG, PTEN, PTGS2, TP53. The results provided a new direction and a theoretical basis for the treatment of periodontitis, but the validation with network pharmacology and molecular docking still had some limitations. Experimental validation of the theoretical results will be the next step in our research.

### Supplementary Information

Supplementary Material 1.

Supplementary Material 2.

## Data Availability

No datasets were generated or analysed during the current study.

## Abbreviations

AKT: Protein kinase B
BP: Biological process
CCL5: C–C chemokine ligand 5
CC: Cellular component
CXCR4: C-X-C chemokine receptor type 4
COX-2: Cyclooxygenase-2
CCL20: Cysteine-cysteine motif chemokine ligand 20
DPSCs: Dental pulp stem cells
EFP: European Federation of Periodobology
hPDLCs: Human periodontal ligament cells
hGF: Human gingival fibroblasts
IL-1β: Interleukin-1β
MMPs: Molecular dynamics simulation
MD: Matrix metalloproteinases
MF: Molecular function
mTOR: Mammalian target of rapamycin
NF-κB: Nuclear factor kappa-B
OPN: Osteopontin
PD: Pocket depth
PPI: Protein–protein interaction
PPARG: Peroxisome proliferator-activated receptor gamma
PTEN: Phosphatase and tensin homolog
PTGS2: Prostaglandin-endoperoxide synthase 2
PI3K: Phosphatidylinositide 3-kinase
SHI: Shikonin
TP53: Cellular tumor antigen p53
TNF-α: Tumor necrosis factor-alpha
