# Supplementary material for: Based on network pharmacology and molecular docking to explore the potential mechanism of shikonin in periodontitis
Source: BMC Oral Health. 2024 Jul 24;24:839. doi: 10.1186/s12903-024-04618-7 (PMC11270799; doi:10.1186/s12903-024-04618-7)
Supplement: Supplementary file 1 — Supplementary Material 1. [file 12903_2024_4618_MOESM1_ESM.docx]

| NO. | Gene names | TCMSP Target | SEA Target |
| --- | --- | --- | --- |
| 1 | PTGS1 | PTGS1 | PTGS1 |
| 2 | ESRRG | ESRRG | PPARG |
| 3 | AR | AR | PTGS2 |
| 4 | PPARG | PPARG | PTEN |
| 5 | PTGS2 | PTGS2 | AKT1 |
| 6 | CA2 | CA2 | CXCR4 |
| 7 | CPHA | CPHA | CCL5 |
| 8 | EP300 | EP300 | TP53 |
| 9 | GCN5 | GCN5 | ALPP |
| 10 | GLD-1 | GLD-1 | ALPL |
| 11 | SRTA | SRTA |  |
| 12 | TYR | TYR |  |
| 13 | PTEN | PTEN |  |
| 14 | AKT1 | AKT1 |  |
| 15 | CXCR4 | CXCR4 |  |
| 16 | CCL5 | CCL5 |  |
| 17 | PKM | PKM |  |
| 18 | TP53 | TP53 |  |
| 19 | ANXA5 | ANXA5 |  |
| 20 | ALPP | ALPP |  |
| 21 | ALPL | ALPL |  |
| 22 | ARNTL | ARNTL |  |

Supplementary Table 1: The 22 target genes of shikonin

(22 target genes were obtained from the TCMSP database and the SEA database retrieved 10 target genes, a total of 22 target genes was identified)
