# Supplementary material for: Based on network pharmacology and molecular docking to explore the potential mechanism of shikonin in periodontitis
Source: BMC Oral Health. 2024 Jul 24;24:839. doi: 10.1186/s12903-024-04618-7 (PMC11270799; doi:10.1186/s12903-024-04618-7)
Supplement: Supplementary file 2 — Supplementary Material 2. [file 12903_2024_4618_MOESM2_ESM.docx]

| NO. | Gene names | GeneCards | drugbank | MIMO |
| --- | --- | --- | --- | --- |
| 1 | CTSC | CTSC | RPSI | IL1A |
| 2 | IL1B | IL1B | RPSD | CTSC |
| 3 | FPR1 | FPR1 | IL1B | NOD1 |
| 4 | C1R | C1R | ALOX5 | GLT6D1 |
| 5 | PDON2 | PDON2 | MMP9 | LTF |
| 6 | C1S | C1S | VEGFA | CDKN2BAS |
| 7 | IL1A | IL1A | CASP1 | C1R |
| 8 | IL6 | IL6 | CASP3 | ESR1 |
| 9 | IL36RN | IL36RN | CYCS | PRTN3 |
| 10 | TNF | TNF | NOS2 | ITGB2 |
| 11 | AP1S3 | AP1S3 | HDC | IL18 |
| 12 | MMP8 | MMP8 | HARS |  |
| 13 | TNFSF11 | TNFSF11 | SLC38A3 |  |
| 14 | MMP1 | MMP1 | HAL |  |
| 15 | LNCPOIR | LNCPOIR |  |  |
| 16 | CXCL8 | CXCL8 |  |  |
| 17 | IL10 | IL10 |  |  |
| 18 | ELANE | ELANE |  |  |
| 19 | CDKN2B-AS1 | CDKN2B-AS1 |  |  |
| 20 | TLR4 | TLR4 |  |  |
| 21 | VDR | VDR |  |  |
| 22 | IL17A | IL17A |  |  |
| 23 | IL4 | IL4 |  |  |
| 24 | TNFRSF11B | TNFRSF11B |  |  |
| 25 | MMP9 | MMP9 |  |  |
| 26 | TLR2 | TLR2 |  |  |
| 27 | BGLAP | BGLAP |  |  |
| 28 | IL1RN | IL1RN |  |  |
| 29 | IFNG | IFNG |  |  |
| 30 | MMP2 | MMP2 |  |  |
| 31 | ITGB2 | ITGB2 |  |  |
| 32 | LTF | LTF |  |  |
| 33 | FCGR3B | FCGR3B |  |  |
| 34 | CRP | CRP |  |  |
| 35 | TIMP1 | TIMP1 |  |  |
| 36 | CTSG | CTSG |  |  |
| 37 | RUNX2 | RUNX2 |  |  |
| 38 | FCGR2A | FCGR2A |  |  |
| 39 | CTSK | CTSK |  |  |
| 40 | CCL2 | CCL2 |  |  |
| 41 | IL18 | IL18 |  |  |
| 42 | FCGR3A | FCGR3A |  |  |
| 43 | DEFB1 | DEFB1 |  |  |
| 44 | IBSP | IBSP |  |  |
| 45 | PTGS2 | PTGS2 |  |  |
| 46 | MMP13 | MMP13 |  |  |
| 47 | MMP3 | MMP3 |  |  |
| 48 | DSPP | DSPP |  |  |
| 49 | ASPN | ASPN |  |  |
| 50 | MPO | MPO |  |  |
| 51 | IL2 | IL2 |  |  |
| 52 | SIGLEC5 | SIGLEC5 |  |  |
| 53 | SELE | SELE |  |  |
| 54 | HLA-B | HLA-B |  |  |
| 55 | TGFB1 | TGFB1 |  |  |
| 56 | POSTN | POSTN |  |  |
| 57 | ICAM1 | ICAM1 |  |  |
| 58 | MIR146A | MIR146A |  |  |
| 59 | CD14 | CD14 |  |  |
| 60 | CD36 | CD36 |  |  |
| 61 | NOD2 | NOD2 |  |  |
| 62 | DEFB4A | DEFB4A |  |  |
| 63 | GLT6D1 | GLT6D1 |  |  |
| 64 | COL1A1 | COL1A1 |  |  |
| 65 | HLA-DRB1 | HLA-DRB1 |  |  |
| 66 | IL13 | IL13 |  |  |
| 67 | COL3A1 | COL3A1 |  |  |
| 68 | SPP1 | SPP1 |  |  |
| 69 | ACP5 | ACP5 |  |  |
| 70 | CCL3 | CCL3 |  |  |
| 71 | MIR125A | MIR125A |  |  |
| 72 | FERMT1 | FERMT1 |  |  |
| 73 | ALPP | ALPP |  |  |
| 74 | BMP2 | BMP2 |  |  |
| 75 | SERPINE1 | SERPINE1 |  |  |
| 76 | NOTCH2 | NOTCH2 |  |  |
| 77 | MIR21 | MIR21 |  |  |
| 78 | CERNA3 | CERNA3 |  |  |
| 79 | DEFB103B | DEFB103B |  |  |
| 80 | VEGFA | VEGFA |  |  |
| 81 | DPP7 | DPP7 |  |  |
| 82 | SIRT1 | SIRT1 |  |  |
| 83 | CAMP | CAMP |  |  |
| 84 | CXCL10 | CXCL10 |  |  |
| 85 | CAT | CAT |  |  |
| 86 | MIR17 | MIR17 |  |  |
| 87 | TGFB2 | TGFB2 |  |  |
| 88 | CEMP1 | CEMP1 |  |  |
| 89 | SP7 | SP7 |  |  |
| 90 | MIR214 | MIR214 |  |  |
| 91 | MIR1226 | MIR1226 |  |  |
| 92 | MIR140 | MIR140 |  |  |
| 93 | SLC17A5 | SLC17A5 |  |  |
| 94 | AMELX | AMELX |  |  |
| 95 | BRINP3 | BRINP3 |  |  |
| 96 | FN1 | FN1 |  |  |
| 97 | FBN2 | FBN2 |  |  |
| 98 | PLAT | PLAT |  |  |
| 99 | CCR6 | CCR6 |  |  |
| 100 | HTN3 | HTN3 |  |  |
| 101 | TIMP2 | TIMP2 |  |  |
| 102 | SLC35C1 | SLC35C1 |  |  |
| 103 | STATH | STATH |  |  |
| 104 | SPARC | SPARC |  |  |
| 105 | CXCR4 | CXCR4 |  |  |
| 106 | H19 | H19 |  |  |
| 107 | COL5A1 | COL5A1 |  |  |
| 108 | ESR1 | ESR1 |  |  |
| 109 | MIR142 | MIR142 |  |  |
| 110 | UNC50 | UNC50 |  |  |
| 111 | SERPINA3 | SERPINA3 |  |  |
| 112 | DANCR | DANCR |  |  |
| 113 | PLG | PLG |  |  |
| 114 | MIR144 | MIR144 |  |  |
| 115 | IGF1 | IGF1 |  |  |
| 116 | BDNF-AS | BDNF-AS |  |  |
| 117 | SELL | SELL |  |  |
| 118 | ALB | ALB |  |  |
| 119 | TGFBR2 | TGFBR2 |  |  |
| 120 | TNXB | TNXB |  |  |
| 121 | COL1A2 | COL1A2 |  |  |
| 122 | SMAD3 | SMAD3 |  |  |
| 123 | FGF2 | FGF2 |  |  |
| 124 | MIF | MIF |  |  |
| 125 | CXCR2 | CXCR2 |  |  |
| 126 | NLRP10 | NLRP10 |  |  |
| 127 | PPARG | PPARG |  |  |
| 128 | SLC23A1 | SLC23A1 |  |  |
| 129 | CASP4 | CASP4 |  |  |
| 130 | SOD2-OT1 | SOD2-OT1 |  |  |
| 131 | PADI4 | PADI4 |  |  |
| 132 | GUSB | GUSB |  |  |
| 133 | CCR5 | CCR5 |  |  |
| 134 | MEG3 | MEG3 |  |  |
| 135 | SLC24A4 | SLC24A4 |  |  |
| 136 | FGF10 | FGF10 |  |  |
| 137 | DCN | DCN |  |  |
| 138 | ENSG00000275307 | ENSG00000275307 |  |  |
| 139 | ENSG00000276965 | ENSG00000276965 |  |  |
| 140 | LYST | LYST |  |  |
| 141 | LINC02605 | LINC02605 |  |  |
| 142 | FCGR2B | FCGR2B |  |  |
| 143 | SERPINB2 | SERPINB2 |  |  |
| 144 | CASP3 | CASP3 |  |  |
| 145 | HLA-DQB1 | HLA-DQB1 |  |  |
| 146 | VCAM1 | VCAM1 |  |  |
| 147 | LPO | LPO |  |  |
| 148 | DLX3 | DLX3 |  |  |
| 149 | CCL21 | CCL21 |  |  |
| 150 | ESR2 | ESR2 |  |  |
| 151 | KLK4 | KLK4 |  |  |
| 152 | ENAM | ENAM |  |  |
| 153 | LTBP3 | LTBP3 |  |  |
| 154 | GPR68 | GPR68 |  |  |
| 155 | GFI1 | GFI1 |  |  |
| 156 | COL17A1 | COL17A1 |  |  |
| 157 | HEY2 | HEY2 |  |  |
| 158 | FAM83H | FAM83H |  |  |
| 159 | CSF1 | CSF1 |  |  |
| 160 | NFATC1 | NFATC1 |  |  |
| 161 | CCL5 | CCL5 |  |  |
| 162 | NLRP3 | NLRP3 |  |  |
| 163 | EMSLR | EMSLR |  |  |
| 164 | MIR493HG | MIR493HG |  |  |
| 165 | ENSG00000276919 | ENSG00000276919 |  |  |
| 166 | HLA-A | HLA-A |  |  |
| 167 | WDR72 | WDR72 |  |  |
| 168 | MIR155 | MIR155 |  |  |
| 169 | MMP12 | MMP12 |  |  |
| 170 | CD79A | CD79A |  |  |
| 171 | LTA | LTA |  |  |
| 172 | HAX1 | HAX1 |  |  |
| 173 | BMP6 | BMP6 |  |  |
| 174 | BMP7 | BMP7 |  |  |
| 175 | AGER | AGER |  |  |
| 176 | MAPK1 | MAPK1 |  |  |
| 177 | CTLA4 | CTLA4 |  |  |
| 178 | EGF | EGF |  |  |
| 179 | CXCL12 | CXCL12 |  |  |
| 180 | TREM1 | TREM1 |  |  |
| 181 | SLC37A4 | SLC37A4 |  |  |
| 182 | ADIPOQ | ADIPOQ |  |  |
| 183 | ITGB6 | ITGB6 |  |  |
| 184 | IL11 | IL11 |  |  |
| 185 | HMGB1 | HMGB1 |  |  |
| 186 | PRTN3 | PRTN3 |  |  |
| 187 | HSPG2 | HSPG2 |  |  |
| 188 | TNFRSF11A | TNFRSF11A |  |  |
| 189 | IL1R1 | IL1R1 |  |  |
| 190 | IFIH1 | IFIH1 |  |  |
| 191 | IL33 | IL33 |  |  |
| 192 | CSF2 | CSF2 |  |  |
| 193 | CSF3 | CSF3 |  |  |
| 194 | HIF1A | HIF1A |  |  |
| 195 | FOS | FOS |  |  |
| 196 | FBN1 | FBN1 |  |  |
| 197 | LINC01672 | LINC01672 |  |  |
| 198 | ELN | ELN |  |  |
| 199 | ITGAL | ITGAL |  |  |
| 200 | AMBN | AMBN |  |  |
| 201 | NFKB1 | NFKB1 |  |  |
| 202 | NOS2 | NOS2 |  |  |
| 203 | IL12B | IL12B |  |  |
| 204 | MBL2 | MBL2 |  |  |
| 205 | PLOD1 | PLOD1 |  |  |
| 206 | ARHGAP6 | ARHGAP6 |  |  |
| 207 | PRDM5 | PRDM5 |  |  |
| 208 | SP6 | SP6 |  |  |
| 209 | ZNF469 | ZNF469 |  |  |
| 210 | LOC101448202 | LOC101448202 |  |  |
| 211 | LOC106780803 | LOC106780803 |  |  |
| 212 | IL34 | IL34 |  |  |
| 213 | ALPL | ALPL |  |  |
| 214 | TMX2-CTNND1 | TMX2-CTNND1 |  |  |
| 215 | ITGAM | ITGAM |  |  |
| 216 | HSPD1 | HSPD1 |  |  |
| 217 | OSM | OSM |  |  |
| 218 | NPY | NPY |  |  |
| 219 | FMOD | FMOD |  |  |
| 220 | JUN | JUN |  |  |
| 221 | MAPK14 | MAPK14 |  |  |
| 222 | RETN | RETN |  |  |
| 223 | S100A8 | S100A8 |  |  |
| 224 | PGR-AS1 | PGR-AS1 |  |  |
| 225 | CTSB | CTSB |  |  |
| 226 | ODAM | ODAM |  |  |
| 227 | IL12A | IL12A |  |  |
| 228 | LBP | LBP |  |  |
| 229 | IL1RAPL2 | IL1RAPL2 |  |  |
| 230 | GSTM1 | GSTM1 |  |  |
| 231 | AMTN | AMTN |  |  |
| 232 | IL17F | IL17F |  |  |
| 233 | TERT | TERT |  |  |
| 234 | DEFA1 | DEFA1 |  |  |
| 235 | HMOX1 | HMOX1 |  |  |
| 236 | CTSL | CTSL |  |  |
| 237 | TP53 | TP53 |  |  |
| 238 | FGB | FGB |  |  |
| 239 | PTHLH | PTHLH |  |  |
| 240 | CD40LG | CD40LG |  |  |
| 241 | CYP1A1 | CYP1A1 |  |  |
| 242 | NAMPT | NAMPT |  |  |
| 243 | MIR223 | MIR223 |  |  |
| 244 | JAG1 | JAG1 |  |  |
| 245 | HGF | HGF |  |  |
| 246 | CTNNB1 | CTNNB1 |  |  |
| 247 | XIST | XIST |  |  |
| 248 | MIRLET7F1 | MIRLET7F1 |  |  |
| 249 | ACE | ACE |  |  |
| 250 | NCF4 | NCF4 |  |  |
| 251 | NCF1 | NCF1 |  |  |
| 252 | NCF4-AS1 | NCF4-AS1 |  |  |
| 253 | LOC106029312 | LOC106029312 |  |  |
| 254 | IL23A | IL23A |  |  |
| 255 | FGFR2 | FGFR2 |  |  |
| 256 | P2RX7 | P2RX7 |  |  |
| 257 | NOTCH3 | NOTCH3 |  |  |
| 258 | ICOSLG | ICOSLG |  |  |
| 259 | CMA1 | CMA1 |  |  |
| 260 | FAM20A | FAM20A |  |  |
| 261 | MMP14 | MMP14 |  |  |
| 262 | LACTB | LACTB |  |  |
| 263 | IL16 | IL16 |  |  |
| 264 | LEP | LEP |  |  |
| 265 | SOD1 | SOD1 |  |  |
| 266 | MAPK8 | MAPK8 |  |  |
| 267 | SLPI | SLPI |  |  |
| 268 | SFTA3 | SFTA3 |  |  |
| 269 | SNRPN | SNRPN |  |  |
| 270 | MIR543 | MIR543 |  |  |
| 271 | AP3B1 | AP3B1 |  |  |
| 272 | S100A9 | S100A9 |  |  |
| 273 | ENSG00000277577 | ENSG00000277577 |  |  |
| 274 | MIR146B | MIR146B |  |  |
| 275 | CCL20 | CCL20 |  |  |
| 276 | IL6R | IL6R |  |  |
| 277 | MMP7 | MMP7 |  |  |
| 278 | TNFRSF1B | TNFRSF1B |  |  |
| 279 | FGD5-AS1 | FGD5-AS1 |  |  |
| 280 | HLA-C | HLA-C |  |  |
| 281 | F2RL1 | F2RL1 |  |  |
| 282 | BSG | BSG |  |  |
| 283 | SNHG14 | SNHG14 |  |  |
| 284 | CST3 | CST3 |  |  |
| 285 | BCL2A1 | BCL2A1 |  |  |
| 286 | NOS3 | NOS3 |  |  |
| 287 | ALDH2 | ALDH2 |  |  |
| 288 | MIR628 | MIR628 |  |  |
| 289 | SERPINA1 | SERPINA1 |  |  |
| 290 | IL1R2 | IL1R2 |  |  |
| 291 | DMP1 | DMP1 |  |  |
| 292 | CD40 | CD40 |  |  |
| 293 | KLK11 | KLK11 |  |  |
| 294 | TLR9 | TLR9 |  |  |
| 295 | S100A4 | S100A4 |  |  |
| 296 | SELP | SELP |  |  |
| 297 | ADAM17 | ADAM17 |  |  |
| 298 | ITGAX | ITGAX |  |  |
| 299 | CXCR1 | CXCR1 |  |  |
| 300 | G6PC1 | G6PC1 |  |  |
| 301 | BDNF | BDNF |  |  |
| 302 | IL4R | IL4R |  |  |
| 303 | DPP4 | DPP4 |  |  |
| 304 | NOTCH1 | NOTCH1 |  |  |
| 305 | FASLG | FASLG |  |  |
| 306 | AKT1 | AKT1 |  |  |
| 307 | PTGS1 | PTGS1 |  |  |
| 308 | GSTT1 | GSTT1 |  |  |
| 309 | TWIST1 | TWIST1 |  |  |
| 310 | FGF3 | FGF3 |  |  |
| 311 | AEBP1 | AEBP1 |  |  |
| 312 | NPAP1 | NPAP1 |  |  |
| 313 | PLAU | PLAU |  |  |
| 314 | MAPK3 | MAPK3 |  |  |
| 315 | YAP1 | YAP1 |  |  |
| 316 | EGFR | EGFR |  |  |
| 317 | PTGER2 | PTGER2 |  |  |
| 318 | GPT | GPT |  |  |
| 319 | FCAR | FCAR |  |  |
| 320 | USB1 | USB1 |  |  |
| 321 | PTK2 | PTK2 |  |  |
| 322 | HTN1 | HTN1 |  |  |
| 323 | CCN2 | CCN2 |  |  |
| 324 | PTGER3 | PTGER3 |  |  |
| 325 | IGF2 | IGF2 |  |  |
| 326 | IL7 | IL7 |  |  |
| 327 | HP | HP |  |  |
| 328 | FAM135B | FAM135B |  |  |
| 329 | PHEX | PHEX |  |  |
| 330 | IGF1R | IGF1R |  |  |
| 331 | TRA-TGC7-1 | TRA-TGC7-1 |  |  |
| 332 | TRA-TGC5-1 | TRA-TGC5-1 |  |  |
| 333 | MIRLET7F2 | MIRLET7F2 |  |  |
| 334 | WNT5A | WNT5A |  |  |
| 335 | PTEN | PTEN |  |  |
| 336 | BIRC5 | BIRC5 |  |  |
| 337 | ITGA4 | ITGA4 |  |  |
| 338 | GDF15 | GDF15 |  |  |
| 339 | BMP4 | BMP4 |  |  |
| 340 | MIR31HG | MIR31HG |  |  |
| 341 | TF | TF |  |  |
| 342 | CD4 | CD4 |  |  |
| 343 | CALCA | CALCA |  |  |
| 344 | CAV1 | CAV1 |  |  |
| 345 | GAA | GAA |  |  |
| 346 | PRKAB2 | PRKAB2 |  |  |
| 347 | MDN1 | MDN1 |  |  |
| 348 | VEZF1 | VEZF1 |  |  |
| 349 | MMP25 | MMP25 |  |  |
| 350 | FIZ1 | FIZ1 |  |  |
| 351 | GREM1 | GREM1 |  |  |
| 352 | DKK1 | DKK1 |  |  |
| 353 | SNHG1 | SNHG1 |  |  |
| 354 | NFE2L2 | NFE2L2 |  |  |
| 355 | EDN1 | EDN1 |  |  |
| 356 | CXCL1 | CXCL1 |  |  |
| 357 | PTGER4 | PTGER4 |  |  |
| 358 | IL5 | IL5 |  |  |
| 359 | TGFBR1 | TGFBR1 |  |  |
| 360 | FBLN5 | FBLN5 |  |  |
| 361 | PDGFB | PDGFB |  |  |
| 362 | ILK | ILK |  |  |
| 363 | PWAR6 | PWAR6 |  |  |
| 364 | FOXP3 | FOXP3 |  |  |
| 365 | LINC01618 | LINC01618 |  |  |
| 366 | MIR24-1 | MIR24-1 |  |  |
| 367 | CTSD | CTSD |  |  |
| 368 | SOD2 | SOD2 |  |  |
| 369 | MIR203A | MIR203A |  |  |
| 370 | SLC52A3 | SLC52A3 |  |  |
| 371 | VPS11 | VPS11 |  |  |
| 372 | SLC52A2 | SLC52A2 |  |  |
| 373 | GDF5 | GDF5 |  |  |
| 374 | MALAT1 | MALAT1 |  |  |
| 375 | LGALS3 | LGALS3 |  |  |
| 376 | PGLYRP1 | PGLYRP1 |  |  |
| 377 | CXCR3 | CXCR3 |  |  |
| 378 | EFEMP2 | EFEMP2 |  |  |
| 379 | TNFRSF1A | TNFRSF1A |  |  |
| 380 | SP1 | SP1 |  |  |
| 381 | MIR23A | MIR23A |  |  |
| 382 | GC | GC |  |  |
| 383 | ADIPOR1 | ADIPOR1 |  |  |
| 384 | ITGB1 | ITGB1 |  |  |
| 385 | LEPR | LEPR |  |  |
| 386 | G6PC3 | G6PC3 |  |  |
| 387 | VTN | VTN |  |  |
| 388 | GNRH1 | GNRH1 |  |  |
| 389 | IFI16 | IFI16 |  |  |
| 390 | MIR150 | MIR150 |  |  |
| 391 | CD44 | CD44 |  |  |
| 392 | NOD1 | NOD1 |  |  |
| 393 | FERMT2 | FERMT2 |  |  |
| 394 | CDKN2A | CDKN2A |  |  |
| 395 | C5AR1 | C5AR1 |  |  |
| 396 | AGTR1 | AGTR1 |  |  |
| 397 | PDGFA | PDGFA |  |  |
| 398 | GZMB | GZMB |  |  |
| 399 | HLA-DRB3 | HLA-DRB3 |  |  |
| 400 | IPW | IPW |  |  |
| 401 | CCL7 | CCL7 |  |  |
| 402 | LAMB3 | LAMB3 |  |  |
| 403 | LAMA3 | LAMA3 |  |  |
| 404 | PIK3CG | PIK3CG |  |  |
| 405 | PLEK | PLEK |  |  |
| 406 | PWAR1 | PWAR1 |  |  |
| 407 | PTGER1 | PTGER1 |  |  |
| 408 | MIR381 | MIR381 |  |  |
| 409 | GHRL | GHRL |  |  |
| 410 | TNFSF13B | TNFSF13B |  |  |
| 411 | COL4A1 | COL4A1 |  |  |
| 412 | PKP2 | PKP2 |  |  |
| 413 | FGF7 | FGF7 |  |  |
| 414 | TGIF1 | TGIF1 |  |  |
| 415 | IL2RA | IL2RA |  |  |
| 416 | SFRP1 | SFRP1 |  |  |
| 417 | FBXO38 | FBXO38 |  |  |
| 418 | LINC01126 | LINC01126 |  |  |
| 419 | NIN | NIN |  |  |
| 420 | NELL1 | NELL1 |  |  |
| 421 | SOST | SOST |  |  |
| 422 | IFNGR1 | IFNGR1 |  |  |
| 423 | EDIL3 | EDIL3 |  |  |
| 424 | MMP19 | MMP19 |  |  |
| 425 | MMP24 | MMP24 |  |  |
| 426 | PUS3 | PUS3 |  |  |
| 427 | PIMREG | PIMREG |  |  |
| 428 | MTFR2 | MTFR2 |  |  |
| 429 | MIR4450 | MIR4450 |  |  |
| 430 | RELA | RELA |  |  |
| 431 | IFNL1 | IFNL1 |  |  |
| 432 | IGSF3 | IGSF3 |  |  |
| 433 | GAS5 | GAS5 |  |  |
| 434 | TLR5 | TLR5 |  |  |
| 435 | KCNQ1OT1 | KCNQ1OT1 |  |  |
| 436 | IL6ST | IL6ST |  |  |
| 437 | APOE | APOE |  |  |
| 438 | INS | INS |  |  |
| 439 | MIR210 | MIR210 |  |  |
| 440 | FNDC5 | FNDC5 |  |  |
| 441 | CST7 | CST7 |  |  |
| 442 | PRSS57 | PRSS57 |  |  |
| 443 | SIGLEC7 | SIGLEC7 |  |  |
| 444 | CNR1 | CNR1 |  |  |
| 445 | TRP-AGG2-5 | TRP-AGG2-5 |  |  |
| 446 | TRP-AGG2-6 | TRP-AGG2-6 |  |  |
| 447 | TRP-AGG2-1 | TRP-AGG2-1 |  |  |
| 448 | TRP-AGG2-2 | TRP-AGG2-2 |  |  |
| 449 | TRP-AGG2-3 | TRP-AGG2-3 |  |  |
| 450 | TRP-AGG2-4 | TRP-AGG2-4 |  |  |
| 451 | TRP-AGG2-7 | TRP-AGG2-7 |  |  |
| 452 | TRP-AGG2-8 | TRP-AGG2-8 |  |  |
| 453 | IL36G | IL36G |  |  |
| 454 | MIAT | MIAT |  |  |
| 455 | TNFSF10 | TNFSF10 |  |  |
| 456 | MIR31 | MIR31 |  |  |
| 457 | PCSK9 | PCSK9 |  |  |
| 458 | MEFV | MEFV |  |  |
| 459 | P2RX5-TAX1BP3 | P2RX5-TAX1BP3 |  |  |
| 460 | FDCSP | FDCSP |  |  |
| 461 | PTGDS | PTGDS |  |  |
| 462 | KRT23 | KRT23 |  |  |
| 463 | MIR671 | MIR671 |  |  |
| 464 | VAMP3 | VAMP3 |  |  |
| 465 | CEBPB | CEBPB |  |  |
| 466 | MIR130A | MIR130A |  |  |
| 467 | HLA-DRB5 | HLA-DRB5 |  |  |
| 468 | HLA-DRB4 | HLA-DRB4 |  |  |
| 469 | FERMT3 | FERMT3 |  |  |
| 470 | CYBA | CYBA |  |  |
| 471 | BCL2 | BCL2 |  |  |
| 472 | MSX2 | MSX2 |  |  |
| 473 | IL12RB2 | IL12RB2 |  |  |
| 474 | TH | TH |  |  |
| 475 | RGS2 | RGS2 |  |  |
| 476 | MEPE | MEPE |  |  |
| 477 | FGF1 | FGF1 |  |  |
| 478 | PCAT1 | PCAT1 |  |  |
| 479 | DEFA3 | DEFA3 |  |  |
| 480 | HEY1 | HEY1 |  |  |
| 481 | ENSG00000261069 | ENSG00000261069 |  |  |
| 482 | THBD | THBD |  |  |
| 483 | CD28 | CD28 |  |  |
| 484 | GHSR | GHSR |  |  |
| 485 | CDKN2B | CDKN2B |  |  |
| 486 | TNFRSF10D | TNFRSF10D |  |  |
| 487 | SFTPD | SFTPD |  |  |
| 488 | PIK3R1 | PIK3R1 |  |  |
| 489 | MIR200B | MIR200B |  |  |
| 490 | WWTR1 | WWTR1 |  |  |
| 491 | SNORD15A | SNORD15A |  |  |
| 492 | ADM | ADM |  |  |
| 493 | MIRLET7C | MIRLET7C |  |  |
| 494 | NPM1 | NPM1 |  |  |
| 495 | TYMS | TYMS |  |  |
| 496 | DKC1 | DKC1 |  |  |
| 497 | TCIRG1 | TCIRG1 |  |  |
| 498 | CLPB | CLPB |  |  |
| 499 | NHP2 | NHP2 |  |  |
| 500 | OCRL | OCRL |  |  |
| 501 | PARN | PARN |  |  |
| 502 | SRP54 | SRP54 |  |  |
| 503 | RTEL1 | RTEL1 |  |  |
| 504 | NOP10 | NOP10 |  |  |
| 505 | TINF2 | TINF2 |  |  |
| 506 | WRAP53 | WRAP53 |  |  |
| 507 | MIA3 | MIA3 |  |  |
| 508 | GORAB | GORAB |  |  |
| 509 | CTC1 | CTC1 |  |  |
| 510 | TERC | TERC |  |  |
| 511 | IL3 | IL3 |  |  |
| 512 | OMP | OMP |  |  |
| 513 | AGT | AGT |  |  |
| 514 | FCER1A | FCER1A |  |  |
| 515 | MYD88 | MYD88 |  |  |
| 516 | ADIPOR2 | ADIPOR2 |  |  |
| 517 | KAT2A | KAT2A |  |  |
| 518 | CDH1 | CDH1 |  |  |
| 519 | IGF2R | IGF2R |  |  |
| 520 | LY96 | LY96 |  |  |
| 521 | IL37 | IL37 |  |  |
| 522 | TGFA | TGFA |  |  |
| 523 | CALCR | CALCR |  |  |
| 524 | TLR1 | TLR1 |  |  |
| 525 | PLAUR | PLAUR |  |  |
| 526 | TRAF6 | TRAF6 |  |  |
| 527 | OIP5-AS1 | OIP5-AS1 |  |  |
| 528 | ANGPT1 | ANGPT1 |  |  |
| 529 | THSD4 | THSD4 |  |  |
| 530 | PECAM1 | PECAM1 |  |  |
| 531 | MIR132 | MIR132 |  |  |
| 532 | MIR23B | MIR23B |  |  |
| 533 | S100A12 | S100A12 |  |  |
| 534 | ADAMTS4 | ADAMTS4 |  |  |
| 535 | ANGPTL4 | ANGPTL4 |  |  |
| 536 | MIR30E | MIR30E |  |  |
| 537 | CDH2 | CDH2 |  |  |
| 538 | NR1I2 | NR1I2 |  |  |
| 539 | ACTA2 | ACTA2 |  |  |
| 540 | BMP1 | BMP1 |  |  |
| 541 | CCR1 | CCR1 |  |  |
| 542 | CTHRC1 | CTHRC1 |  |  |
| 543 | ITLN1 | ITLN1 |  |  |
| 544 | MIR379 | MIR379 |  |  |
| 545 | HAS2-AS1 | HAS2-AS1 |  |  |
| 546 | CP | CP |  |  |
| 547 | LPL | LPL |  |  |
| 548 | C5 | C5 |  |  |
| 549 | TIMP3 | TIMP3 |  |  |
| 550 | LINC-ROR | LINC-ROR |  |  |
| 551 | FBLIM1 | FBLIM1 |  |  |
| 552 | SEMA3A | SEMA3A |  |  |
| 553 | SMAD5-AS1 | SMAD5-AS1 |  |  |
| 554 | IGFBP5 | IGFBP5 |  |  |
| 555 | CST1 | CST1 |  |  |
| 556 | LINC01133 | LINC01133 |  |  |
| 557 | CXCL6 | CXCL6 |  |  |
| 558 | SLC6A4 | SLC6A4 |  |  |
| 559 | PRRX2 | PRRX2 |  |  |
| 560 | TFF1 | TFF1 |  |  |
| 561 | SPON1 | SPON1 |  |  |
| 562 | RHOA | RHOA |  |  |
| 563 | CASP7 | CASP7 |  |  |
| 564 | MIR498 | MIR498 |  |  |
| 565 | ADRB3 | ADRB3 |  |  |
| 566 | CDKN3 | CDKN3 |  |  |
| 567 | ACTN1 | ACTN1 |  |  |
| 568 | BLK | BLK |  |  |
| 569 | PIK3C3 | PIK3C3 |  |  |
| 570 | ABCA1 | ABCA1 |  |  |
| 571 | SCN2A | SCN2A |  |  |
| 572 | EPHA3 | EPHA3 |  |  |
| 573 | KDM4B | KDM4B |  |  |
| 574 | ACTN2 | ACTN2 |  |  |
| 575 | CAMK4 | CAMK4 |  |  |
| 576 | CHD1 | CHD1 |  |  |
| 577 | GRIK1 | GRIK1 |  |  |
| 578 | ITGA8 | ITGA8 |  |  |
| 579 | NLGN1 | NLGN1 |  |  |
| 580 | CDH13 | CDH13 |  |  |
| 581 | FOXA1 | FOXA1 |  |  |
| 582 | ROBO2 | ROBO2 |  |  |
| 583 | SMURF2 | SMURF2 |  |  |
| 584 | MTHFS | MTHFS |  |  |
| 585 | PDCD6IP | PDCD6IP |  |  |
| 586 | PTPRT | PTPRT |  |  |
| 587 | RGMA | RGMA |  |  |
| 588 | SUMF1 | SUMF1 |  |  |
| 589 | ETS2 | ETS2 |  |  |
| 590 | FRG1 | FRG1 |  |  |
| 591 | IQSEC1 | IQSEC1 |  |  |
| 592 | LRP12 | LRP12 |  |  |
| 593 | RYR3 | RYR3 |  |  |
| 594 | ERGIC1 | ERGIC1 |  |  |
| 595 | ETNK2 | ETNK2 |  |  |
| 596 | HLA-DOA | HLA-DOA |  |  |
| 597 | LRP1B | LRP1B |  |  |
| 598 | MBD2 | MBD2 |  |  |
| 599 | RBFOX1 | RBFOX1 |  |  |
| 600 | SEMA6A | SEMA6A |  |  |
| 601 | SETMAR | SETMAR |  |  |
| 602 | SS18 | SS18 |  |  |
| 603 | ERC2 | ERC2 |  |  |
| 604 | HS6ST2 | HS6ST2 |  |  |
| 605 | SNTB1 | SNTB1 |  |  |
| 606 | GPN1 | GPN1 |  |  |
| 607 | KCNJ16 | KCNJ16 |  |  |
| 608 | NMUR2 | NMUR2 |  |  |
| 609 | RIT2 | RIT2 |  |  |
| 610 | WDR36 | WDR36 |  |  |
| 611 | WDR73 | WDR73 |  |  |
| 612 | ADAMTS15 | ADAMTS15 |  |  |
| 613 | CSMD1 | CSMD1 |  |  |
| 614 | PSMA8 | PSMA8 |  |  |
| 615 | VPREB1 | VPREB1 |  |  |
| 616 | HYCC1 | HYCC1 |  |  |
| 617 | MFSD1 | MFSD1 |  |  |
| 618 | PARP15 | PARP15 |  |  |
| 619 | PGPEP1 | PGPEP1 |  |  |
| 620 | C1orf87 | C1orf87 |  |  |
| 621 | NRSN1 | NRSN1 |  |  |
| 622 | BCORL1 | BCORL1 |  |  |
| 623 | ZNF385D | ZNF385D |  |  |
| 624 | ETAA1 | ETAA1 |  |  |
| 625 | CCDC13 | CCDC13 |  |  |
| 626 | DAOA | DAOA |  |  |
| 627 | NKAIN2 | NKAIN2 |  |  |
| 628 | RAB6C | RAB6C |  |  |
| 629 | PLEKHG7 | PLEKHG7 |  |  |
| 630 | FAM47A | FAM47A |  |  |
| 631 | HMX3 | HMX3 |  |  |
| 632 | NKAIN3 | NKAIN3 |  |  |
| 633 | PRB2 | PRB2 |  |  |
| 634 | ZNF579 | ZNF579 |  |  |
| 635 | FAM180A | FAM180A |  |  |
| 636 | ZNF524 | ZNF524 |  |  |
| 637 | ST20 | ST20 |  |  |
| 638 | LINC02870 | LINC02870 |  |  |
| 639 | ST20-MTHFS | ST20-MTHFS |  |  |
| 640 | LINC00208 | LINC00208 |  |  |
| 641 | IGL | IGL |  |  |
| 642 | LINC00907 | LINC00907 |  |  |
| 643 | ROCK1P1 | ROCK1P1 |  |  |
| 644 | RYR3-DT | RYR3-DT |  |  |
| 645 | LINC01811 | LINC01811 |  |  |
| 646 | ETS2-AS1 | ETS2-AS1 |  |  |
| 647 | NPM1P2 | NPM1P2 |  |  |
| 648 | CNR2 | CNR2 |  |  |
| 649 | MYC | MYC |  |  |
| 650 | TRPA1 | TRPA1 |  |  |
| 651 | VCAN | VCAN |  |  |
| 652 | PLTP | PLTP |  |  |
| 653 | RNA18SN1 | RNA18SN1 |  |  |
| 654 | SOS2 | SOS2 |  |  |
| 655 | GPD1L | GPD1L |  |  |
| 656 | LAMA2 | LAMA2 |  |  |
| 657 | MAP4K5 | MAP4K5 |  |  |
| 658 | STT3B | STT3B |  |  |
| 659 | SEL1L | SEL1L |  |  |
| 660 | ATL1 | ATL1 |  |  |
| 661 | L2HGDH | L2HGDH |  |  |
| 662 | RAB28 | RAB28 |  |  |
| 663 | CDKL1 | CDKL1 |  |  |
| 664 | FHOD3 | FHOD3 |  |  |
| 665 | NKX3-2 | NKX3-2 |  |  |
| 666 | ARHGAP18 | ARHGAP18 |  |  |
| 667 | CMTM8 | CMTM8 |  |  |
| 668 | SAV1 | SAV1 |  |  |
| 669 | OSBPL10 | OSBPL10 |  |  |
| 670 | DMAC2L | DMAC2L |  |  |
| 671 | TPGS2 | TPGS2 |  |  |
| 672 | KIAA1328 | KIAA1328 |  |  |
| 673 | ZNF860 | ZNF860 |  |  |
| 674 | NEAT1 | NEAT1 |  |  |
| 675 | HSP90AB2P | HSP90AB2P |  |  |
| 676 | LOC102723409 | LOC102723409 |  |  |
| 677 | GOT1 | GOT1 |  |  |
| 678 | MIR30B | MIR30B |  |  |
| 679 | MIR30A | MIR30A |  |  |
| 680 | GGT1 | GGT1 |  |  |
| 681 | MIR199A2 | MIR199A2 |  |  |
| 682 | MIR199A1 | MIR199A1 |  |  |
| 683 | CD63 | CD63 |  |  |
| 684 | CREB1 | CREB1 |  |  |
| 685 | MIR28 | MIR28 |  |  |
| 686 | MIR99A | MIR99A |  |  |
| 687 | MIR9-1 | MIR9-1 |  |  |
| 688 | HSPA5 | HSPA5 |  |  |
| 689 | CCL11 | CCL11 |  |  |
| 690 | GSR | GSR |  |  |
| 691 | KNG1 | KNG1 |  |  |
| 692 | TUG1 | TUG1 |  |  |
| 693 | MEG8 | MEG8 |  |  |
| 694 | FAS | FAS |  |  |
| 695 | ATF4 | ATF4 |  |  |
| 696 | EGLN1 | EGLN1 |  |  |
| 697 | SOCS3 | SOCS3 |  |  |
| 698 | RECK | RECK |  |  |
| 699 | ZEB2 | ZEB2 |  |  |
| 700 | MIR27A | MIR27A |  |  |
| 701 | GSK3B | GSK3B |  |  |
| 702 | SERPINH1 | SERPINH1 |  |  |
| 703 | HSPB1 | HSPB1 |  |  |
| 704 | KRT18 | KRT18 |  |  |
| 705 | CCR4 | CCR4 |  |  |
| 706 | SMAD1 | SMAD1 |  |  |
| 707 | MIR126 | MIR126 |  |  |
| 708 | LIPA | LIPA |  |  |
| 709 | IL22 | IL22 |  |  |
| 710 | HSP90AA1 | HSP90AA1 |  |  |
| 711 | MIR1306 | MIR1306 |  |  |
| 712 | KCNQ5 | KCNQ5 |  |  |
| 713 | NME8 | NME8 |  |  |
| 714 | CRACR2A | CRACR2A |  |  |
| 715 | GPR141 | GPR141 |  |  |
| 716 | PADI2 | PADI2 |  |  |
| 717 | SOCS1 | SOCS1 |  |  |
| 718 | PDCD1 | PDCD1 |  |  |
| 719 | HAS2 | HAS2 |  |  |
| 720 | MK280073-487 | MK280073-487 |  |  |
| 721 | TNN | TNN |  |  |
| 722 | IDO1 | IDO1 |  |  |
| 723 | CD274 | CD274 |  |  |
| 724 | GLDC | GLDC |  |  |
| 725 | PKN2 | PKN2 |  |  |
| 726 | TRPS1 | TRPS1 |  |  |
| 727 | HTR4 | HTR4 |  |  |
| 728 | KCNK1 | KCNK1 |  |  |
| 729 | FZD8 | FZD8 |  |  |
| 730 | GRID1 | GRID1 |  |  |
| 731 | CAMTA1 | CAMTA1 |  |  |
| 732 | CLIC5 | CLIC5 |  |  |
| 733 | JDP2 | JDP2 |  |  |
| 734 | OTOF | OTOF |  |  |
| 735 | TBC1D1 | TBC1D1 |  |  |
| 736 | WWC1 | WWC1 |  |  |
| 737 | DAB2IP | DAB2IP |  |  |
| 738 | UHRF2 | UHRF2 |  |  |
| 739 | WAPL | WAPL |  |  |
| 740 | ANKRD30A | ANKRD30A |  |  |
| 741 | CSMD3 | CSMD3 |  |  |
| 742 | MAP3K21 | MAP3K21 |  |  |
| 743 | TENM2 | TENM2 |  |  |
| 744 | DEFA4 | DEFA4 |  |  |
| 745 | LNPK | LNPK |  |  |
| 746 | PTTG2 | PTTG2 |  |  |
| 747 | TTLL11 | TTLL11 |  |  |
| 748 | TPD52L3 | TPD52L3 |  |  |
| 749 | CIB4 | CIB4 |  |  |
| 750 | EVX2 | EVX2 |  |  |
| 751 | ADGRG6 | ADGRG6 |  |  |
| 752 | HOTAIRM1 | HOTAIRM1 |  |  |
| 753 | EPO | EPO |  |  |
| 754 | KRT1 | KRT1 |  |  |
| 755 | IL24 | IL24 |  |  |
| 756 | ICOS | ICOS |  |  |
| 757 | MIR182 | MIR182 |  |  |
| 758 | GAPDH | GAPDH |  |  |
| 759 | MIR125B1 | MIR125B1 |  |  |
| 760 | PTX3 | PTX3 |  |  |
| 761 | MYLK | MYLK |  |  |
| 762 | LOC126806446 | LOC126806446 |  |  |
| 763 | DLG2 | DLG2 |  |  |
| 764 | SLC1A3-AS1 | SLC1A3-AS1 |  |  |
| 765 | MIR22HG | MIR22HG |  |  |
| 766 | H3C14 | H3C14 |  |  |
| 767 | MIR383 | MIR383 |  |  |
| 768 | PDGFRB | PDGFRB |  |  |
| 769 | GRN | GRN |  |  |
| 770 | IGFBP3 | IGFBP3 |  |  |
| 771 | IL17RA | IL17RA |  |  |
| 772 | LYZ | LYZ |  |  |
| 773 | SCN4A | SCN4A |  |  |
| 774 | CCR2 | CCR2 |  |  |
| 775 | GH-LCR | GH-LCR |  |  |
| 776 | CYP2E1 | CYP2E1 |  |  |
| 777 | GSTP1 | GSTP1 |  |  |
| 778 | HRH1 | HRH1 |  |  |
| 779 | TUFT1 | TUFT1 |  |  |
| 780 | BMP8A | BMP8A |  |  |
| 781 | LCN2 | LCN2 |  |  |
| 782 | GAD2 | GAD2 |  |  |
| 783 | PF4 | PF4 |  |  |
| 784 | CCND2 | CCND2 |  |  |
| 785 | DELEC1 | DELEC1 |  |  |
| 786 | TMSB4X | TMSB4X |  |  |
| 787 | MIR148A | MIR148A |  |  |
| 788 | RAB27A | RAB27A |  |  |
| 789 | TGM1 | TGM1 |  |  |
| 790 | SNORD80 | SNORD80 |  |  |
| 791 | SIRT6 | SIRT6 |  |  |
| 792 | RARRES2 | RARRES2 |  |  |
| 793 | MMP26 | MMP26 |  |  |
| 794 | DCST1 | DCST1 |  |  |
| 795 | POU5F1 | POU5F1 |  |  |
| 796 | ADAM28 | ADAM28 |  |  |
| 797 | MIR3198-1 | MIR3198-1 |  |  |
| 798 | MIR3198-2 | MIR3198-2 |  |  |
| 799 | MTOR | MTOR |  |  |
| 800 | SNHG5 | SNHG5 |  |  |
| 801 | CSN1S1 | CSN1S1 |  |  |
| 802 | ESRRA | ESRRA |  |  |
| 803 | PELP1 | PELP1 |  |  |
| 804 | PROM1 | PROM1 |  |  |
| 805 | THBS1 | THBS1 |  |  |
| 806 | RPS27 | RPS27 |  |  |
| 807 | GATA3 | GATA3 |  |  |
| 808 | RIPK2 | RIPK2 |  |  |
| 809 | CHRNA7 | CHRNA7 |  |  |
| 810 | EGR1 | EGR1 |  |  |
| 811 | LGALS1 | LGALS1 |  |  |
| 812 | SPRY1 | SPRY1 |  |  |
| 813 | ITGA6 | ITGA6 |  |  |
| 814 | HACD1 | HACD1 |  |  |
| 815 | TGFBI | TGFBI |  |  |
| 816 | SOS1 | SOS1 |  |  |
| 817 | H2AC18 | H2AC18 |  |  |
| 818 | RAC2 | RAC2 |  |  |
| 819 | DOCK8 | DOCK8 |  |  |
| 820 | SBDS | SBDS |  |  |
| 821 | DNM1L | DNM1L |  |  |
| 822 | NRP1 | NRP1 |  |  |
| 823 | CTSS | CTSS |  |  |
| 824 | CX3CL1 | CX3CL1 |  |  |
| 825 | VEGFB | VEGFB |  |  |
| 826 | SOCS6 | SOCS6 |  |  |
| 827 | PON1 | PON1 |  |  |
| 828 | ENSG00000232995 | ENSG00000232995 |  |  |
| 829 | SERPINF1 | SERPINF1 |  |  |
| 830 | MIRLET7A1 | MIRLET7A1 |  |  |
| 831 | MIR20A | MIR20A |  |  |
| 832 | MIR520D | MIR520D |  |  |
| 833 | TIMP4 | TIMP4 |  |  |
| 834 | C4A | C4A |  |  |
| 835 | BPI | BPI |  |  |
| 836 | DSG1 | DSG1 |  |  |
| 837 | FGL2 | FGL2 |  |  |
| 838 | FCGR2C | FCGR2C |  |  |
| 839 | MIR16-1 | MIR16-1 |  |  |
| 840 | MIR16-2 | MIR16-2 |  |  |
| 841 | HTRA1 | HTRA1 |  |  |
| 842 | CX3CR1 | CX3CR1 |  |  |
| 843 | EBI3 | EBI3 |  |  |
| 844 | MIR29B1 | MIR29B1 |  |  |
| 845 | LINC00687 | LINC00687 |  |  |
| 846 | CD248 | CD248 |  |  |
| 847 | PIEZO1 | PIEZO1 |  |  |
| 848 | MIR511 | MIR511 |  |  |
| 849 | MTHFR | MTHFR |  |  |
| 850 | CFH | CFH |  |  |
| 851 | ITGA2 | ITGA2 |  |  |
| 852 | ADCY10 | ADCY10 |  |  |
| 853 | CD80 | CD80 |  |  |
| 854 | NLRC5 | NLRC5 |  |  |
| 855 | TNFSF12 | TNFSF12 |  |  |
| 856 | FBXO5 | FBXO5 |  |  |
| 857 | MIR200A | MIR200A |  |  |
| 858 | MIR222 | MIR222 |  |  |
| 859 | MDH2 | MDH2 |  |  |
| 860 | DUSP1 | DUSP1 |  |  |
| 861 | VAV1 | VAV1 |  |  |
| 862 | NCR2 | NCR2 |  |  |
| 863 | ADGRE1 | ADGRE1 |  |  |
| 864 | CDC42EP2 | CDC42EP2 |  |  |
| 865 | CCL18 | CCL18 |  |  |
| 866 | CD19 | CD19 |  |  |
| 867 | FFAR1 | FFAR1 |  |  |
| 868 | MIR320D1 | MIR320D1 |  |  |
| 869 | MIR320D2 | MIR320D2 |  |  |
| 870 | MIR5571 | MIR5571 |  |  |
| 871 | MIR18A | MIR18A |  |  |
| 872 | MIR34A | MIR34A |  |  |
| 873 | GJA1 | GJA1 |  |  |
| 874 | TFRC | TFRC |  |  |
| 875 | ITGA1 | ITGA1 |  |  |
| 876 | MIR320A | MIR320A |  |  |
| 877 | TRPV4 | TRPV4 |  |  |
| 878 | FTH1 | FTH1 |  |  |
| 879 | FTL | FTL |  |  |
| 880 | KDM6B | KDM6B |  |  |
| 881 | AQP3 | AQP3 |  |  |
| 882 | DLX5 | DLX5 |  |  |
| 883 | SEMA3D | SEMA3D |  |  |
| 884 | TNMD | TNMD |  |  |
| 885 | MIR25 | MIR25 |  |  |
| 886 | ST8SIA6-AS1 | ST8SIA6-AS1 |  |  |
| 887 | MME | MME |  |  |
| 888 | MYH11 | MYH11 |  |  |
| 889 | ANTXR2 | ANTXR2 |  |  |
| 890 | TRIM25 | TRIM25 |  |  |
| 891 | KDR | KDR |  |  |
| 892 | LRG1 | LRG1 |  |  |
| 893 | KDM1A | KDM1A |  |  |
| 894 | ST8SIA1 | ST8SIA1 |  |  |
| 895 | ANGPTL2 | ANGPTL2 |  |  |
| 896 | MIR99B | MIR99B |  |  |
| 897 | OCA2 | OCA2 |  |  |
| 898 | HPS3 | HPS3 |  |  |
| 899 | METTL25B | METTL25B |  |  |
| 900 | THNSL2 | THNSL2 |  |  |
| 901 | CHI3L1 | CHI3L1 |  |  |
| 902 | IL1RL1 | IL1RL1 |  |  |
| 903 | ECE1 | ECE1 |  |  |
| 904 | HSPA4 | HSPA4 |  |  |
| 905 | COL5A2 | COL5A2 |  |  |
| 906 | P3H4 | P3H4 |  |  |
| 907 | EZH2 | EZH2 |  |  |
| 908 | ACVR2B | ACVR2B |  |  |
| 909 | LBR | LBR |  |  |
| 910 | NTF4 | NTF4 |  |  |
| 911 | BHLHE40 | BHLHE40 |  |  |
| 912 | CKAP2L | CKAP2L |  |  |
| 913 | BEGAIN | BEGAIN |  |  |
| 914 | UBE3D | UBE3D |  |  |
| 915 | MIR22 | MIR22 |  |  |
| 916 | PTCSC3 | PTCSC3 |  |  |
| 917 | MAP2K1 | MAP2K1 |  |  |
| 918 | CTRL | CTRL |  |  |
| 919 | TLR10 | TLR10 |  |  |
| 920 | DLEU2 | DLEU2 |  |  |
| 921 | ODC1 | ODC1 |  |  |
| 922 | AHSG | AHSG |  |  |
| 923 | PLXNC1 | PLXNC1 |  |  |
| 924 | HNF4A | HNF4A |  |  |
| 925 | PWRN1 | PWRN1 |  |  |
| 926 | ENG | ENG |  |  |
| 927 | ITGA11 | ITGA11 |  |  |
| 928 | MIR301A | MIR301A |  |  |
| 929 | LAMC2 | LAMC2 |  |  |
| 930 | SOX9 | SOX9 |  |  |
| 931 | SMURF1 | SMURF1 |  |  |
| 932 | MCRS1 | MCRS1 |  |  |
| 933 | MIR7-1 | MIR7-1 |  |  |
| 934 | CYCS | CYCS |  |  |
| 935 | TNFAIP3 | TNFAIP3 |  |  |
| 936 | RPSI |  |  |  |
| 937 | RPSD |  |  |  |
| 938 | ALOX5 |  |  |  |
| 939 | CASP1 |  |  |  |
| 940 | HDC |  |  |  |
| 941 | HARS |  |  |  |
| 942 | SLC38A3 |  |  |  |
| 943 | HAL |  |  |  |
| 944 | CDKN2BAS |  |  |  |

Supplementary Table 2: The 944 genes were associated with periodontitis

(Target genes of periodontitis were gathered from three databases, 11 in OMIM, 935 in GeneCards Database and 14 in Drugbank Database. A total of 944 gene targets were identified in periodontitis .)
